# Membrane particles generated from mesenchymal stromal cells modulate immune responses by selective targeting of pro-inflammatory monocytes

**DOI:** 10.1038/s41598-017-12121-z

**Published:** 2017-09-21

**Authors:** Fabiany da C. Gonçalves, Franka Luk, Sander S. Korevaar, Rachid Bouzid, Ana H. Paz, Carmen López-Iglesias, Carla C. Baan, Ana Merino, Martin J. Hoogduijn

**Affiliations:** 1 Graduate Program in Gastroenterology and Hepatology Sciences, Universidade Federal do Rio Grande do Sul, Porto Alegre, RS Brazil; 2 Experimental Research Center, Hospital de Clínicas de Porto Alegre, Porto Alegre, RS Brazil; 3 Nephrology and Transplantation, Internal Medicine, Erasmus Medical Center, Rotterdam, The Netherlands; 4 The Institute of Nanoscopy, Maastricht University, Maastricht, The Netherlands

## Abstract

Mesenchymal stromal cells (MSC) are a promising therapy for immunological disorders. However, culture expanded MSC are large and get trapped in the capillary networks of the lungs after intravenous infusion, where they have a short survival time. Hypothetically, living cells are a risk for tumor formation. To reduce risks associated with MSC infusion and improve the distribution in the body, we generated membrane particles (MP) of MSC and MSC stimulated with IFN-γ (MPγ). Tracking analysis and electron microscopy indicated that the average size of MP was 120 nm, and they showed a round shape. MP exhibited ATPase, nucleotidase and esterase activity, indicating they are enzymatically active. MP and MPγ did not physically interact with T cells and had no effect on CD4^+^ and CD8^+^ T cells proliferation. However, MP and MPγ selectively bound to monocytes and decreased the frequency of pro-inflammatory CD14^+^CD16^+^ monocytes by induction of selective apoptosis. MP and MPγ increased the percentage of CD90 positive monocytes, and MPγ but not MP increased the percentage of anti-inflammatory PD-L1 monocytes. MPγ increased mRNA expression of PD-L1 in monocytes. These data demonstrate that MP have immunomodulatory properties and have potential as a novel cell-free therapy for treatment of immunological disorders.

## Introduction

Mesenchymal stromal cells (MSC) are studied as an experimental therapy for immunological disorders due to their diverse immunomodulatory properties^1–3^. Multiple clinical trials with MSC in inflammatory disease and transplantation have been conducted, such as in graft versus host disease^4^, kidney transplantation^5^, and Crohn’s disease^6^. The outcomes of several of these trials hint towards a beneficial immunomodulatory effect of MSC, but they are not conclusive^7^. This is partly due to the small patient numbers, to the lack of understanding of the effects of MSC after administration, and perhaps because MSC derived from different tissue sources are used which display distinct paracrine potential and immune regulatory properties. Several authors have compared the capacity of MSC from various tissue sources to suppress peripheral blood B, T and NK cells, and it has been reported that adipose tissue-derived MSC (AT-MSC) have a stronger immunomodulatory effect than MSC from other tissue sources^8,9^. The function of MSC as immunomodulatory agent has been attributed to a variety of mechanisms, notably cytokine and chemokine secretion^10,11^. Multiple pathways have been identified to play a role in *in vitro* assays, but it is unknown whether they play a role in the immunomodulatory effects of MSC administered to animals or patients. Intravenous infusion has been used as the route of MSC delivery for most preclinical studies^12,13^ and clinical trials^7^. It was the assumption that intravenous infusion of MSC would lead to a broad bio-distribution of MSC. However, tracking studies have shown that the majority of MSC localize to the lungs after intravenous infusion. The detainment of MSC in the lungs is due to their size (>20 μm in diameter)^14,15^, which exceeds the width of the micro-capillaries of the lungs. It has furthermore become clear that MSC have a short-term survival after infusion^16,17^. Over 90% of infused MSC are lost within 24 h after infusion. Even though infused MSC end up in the lungs and disappear rapidly, they exert immunomodulatory effects. The short lifespan of MSC after intravenous infusion questions the contribution of secreted anti-inflammatory factors by MSC to the modulation of immune responses.

Recent work demonstrated that heat inactivated MSC that lost their capacity to secrete factors maintain their immunomodulatory capacity after intravenous infusion in an LPS-induced sepsis model, suggesting that cell membrane dependent interactions with immune cells are responsible for the immune regulatory effects^18^. MSC express immunomodulatory molecules on their membrane such as Toll-like receptors (TLRs)^19^, ATPases^20^ and CD73 (ecto-5′-nucleotidase, Ecto5′NTase) which dephosphorylate ATP into AMP and AMP into adenosine, respectively^21^. This is an important immunomodulatory function as adenosine has immunosuppressive properties^22^. MSC also express receptors involved in differentiation pathways such as CD90 (Thy-1 membrane glycoprotein) that is known for its participation on the differentiation of MSC by acting as an obstacle in the pathway of differentiation commitment^23^. The ability of MSC to modulate the immune system can be enhanced by treatment of MSC with pro-inflammatory cytokines, in particular interferon-γ (IFN-γ) and tumor necrosis factor (TNF)-α^24–26^. Under inflammatory conditions MSC upregulate the expression of cell surface proteins with immune regulatory function, such as programmed death ligand 1 (PD-L1), and Fas ligand via which they directly target immune cells and inhibit their activation and function^27^.

Despite of the great potential, several factors including the practical difficulties that come with the use of living cells, their short survival after intravenous infusion and their poor biodistribution, have been major technical challenges to be overcome before MSC based therapy can be used for clinical application in a consistently therapeutic manner^28^. A modification in the treatment that avoids these complications but preserves the diverse immunoregulatory properties of MSC would therefore improve the applicability of this therapy. We propose a new cell-free therapy based on the generation of small plasma membrane particles (MP) from AT-MSC cultured under different conditions. Therefore, the aim of this study was to generate and characterize MP derived from MSC cultured with and without IFN-γ, analyze their immunomodulatory properties, and their interaction with the immune system.

## Results

### Characterization of adipose tissue derived mesenchymal stromal cells

**Figure 1:**
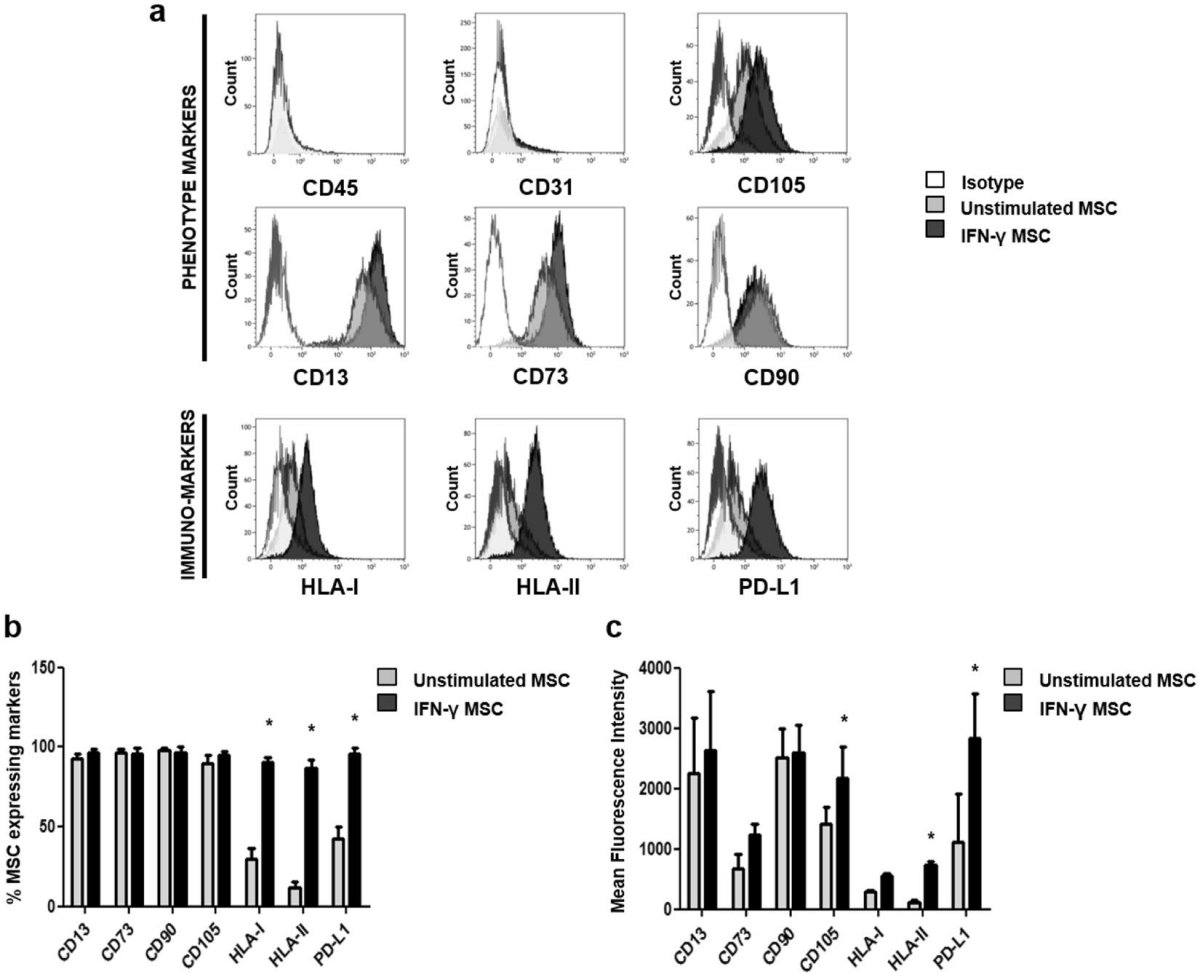
Immunophenotype of unstimulated and IFN-γ stimulated AT-MSC. (**a**) Representative flow cytometry analysis of the commonly used markers for MSC (CD45 and CD31, both negative, and CD105, CD13, CD73, CD90), and the immune-markers HLA-I, HLA-II, and PD-L1. Isotype (white histograms), unstimulated AT-MSC (grey histograms) and IFN-γ AT-﻿MSC (black histograms). (**b**) Percentage positive cells and (**c**) Mean fluorescence intensities (MFI) of the markers on unstimulated and IFN-γ stimulated AT-﻿MSC. Data are presented as mean ± SD from 5 independent experiments. P values refer to the condition without IFN-γ. Unpaired t-test was used for statistical analysis.

Commonly used AT-MSC surface markers were analyzed in unstimulated and IFN-γ stimulated AT-MSC by flow cytometry (Fig. 1a). Both types of cells were negative for the markers CD45 and CD31, and positive for CD13, CD73, CD90 and CD105. There was no statistical significant difference in the percentage of unstimulated and IFN-γ stimulated AT-MSC expressing these markers. However, stimulation with IFN-γ significantly increased the percentage of cells positive for immune-markers such as HLA-I, HLA-II, and PD-L1 (Fig. 1b). The mean fluorescence intensity of the various markers was determined and a significant increase in the expression of CD105, HLA-II, and PD-L1 was observed after IFN-γ treatment (Fig. 1c).

### Generation and characterization of Membrane Particles (MP)

**Figure 2:**
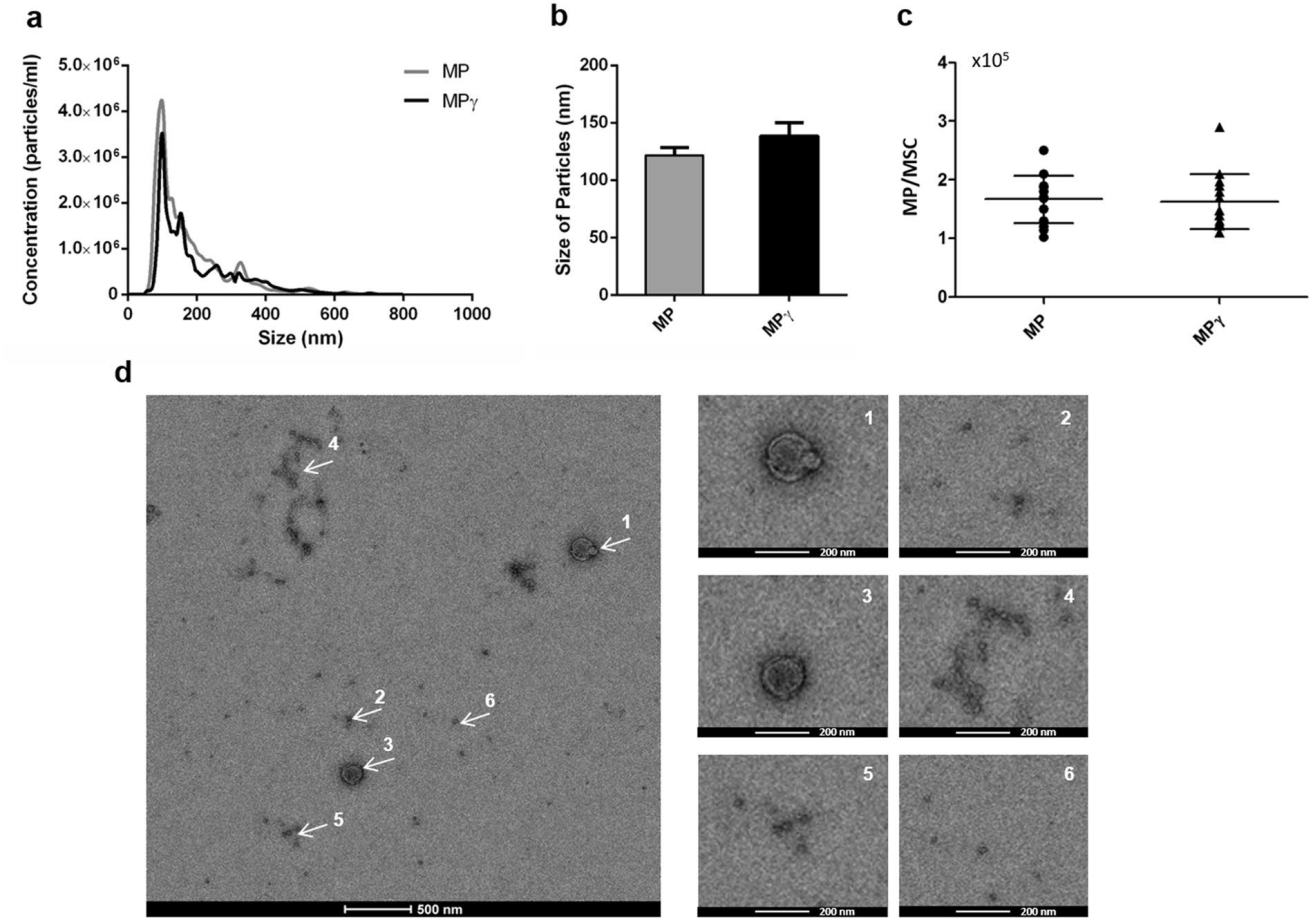
Characterization of Membrane Particles generated from unstimulated and IFN-γ stimulated AT-MSC (MP and MPγ, respectively). (**a**) Nanoparticle tracking analysis (NTA) profiles of MP and MPγ. The NTA software generates a distribution graph on a particle-by-particle basis, a count (in terms of absolute number and concentration), and (**b**) size distribution of MP and MPγ. (**c**) The average number of particles generated per MSC. Data are presented as mean ± SD from 10 independent preparations of MP. There was no statistical difference with respect to concentration and size between MP and MPγ. The statistic test used was unpaired t-test. (**d**) Transmission electron microscopy analysis of MP. White arrows point to areas zoomed in on at the images on the right side. Most of the MP showed a round shape and a size below 200 nm.

MP were generated from unstimulated and IFN-γ stimulated AT-MSC. The number of cells used for each analysis was between 1 × 10^6^–1.5 × 10^6^ cells (80% confluency). The size distribution of the obtained MP was studied using Nanoparticle tracking analysis (NTA). The size of the particles ranged from 63 to 700 nm (Fig. 2a), and the mode size of the samples was 121.7 ± 35.5 nm for MP and 138.3 ± 62.1 nm for MPγ (Fig. 2b). The percentage of particles with a size larger than 200 nm was lower than 5% in every sample.

Based on the particle concentration per ml, the average number of particles generated from each MSC was 1.2 × 10^5^ ± 2.7 × 10^4^ for MP and 1.1 × 10^5^ ± 2.8 × 10^4^ for MPγ (Fig. 2c). There was no significant difference in size distribution or concentration (MP/MSC) between MP and MPγ.

The transmission electron microscopy images illustrate that MP consist of a population of particles heterogeneous in size with most of the particles showing a size of less than 200 nm (Fig. 2d) but some showing larger sizes. This result confirms the NTA analysis. It can be clearly observed that both the larger and smaller MP have a round shape.

### Membrane Particles from AT-MSC possess enzyme activity

To analyze whether MP have enzyme activity, we examined the ability of MP and MPγ to convert ATP to ADP by ATPase activity, and AMP to adenosine by the nucleotidase activity of CD73. The last product of these two reactions is free phosphate, so the samples for these assays were prepared in milliQ water to avoid contamination with free phosphates from saline buffers. Before measurement of enzyme activities, MP (diluted in milliQ water) were analyzed by NTA for determination of their concentration.

**Figure 3:**
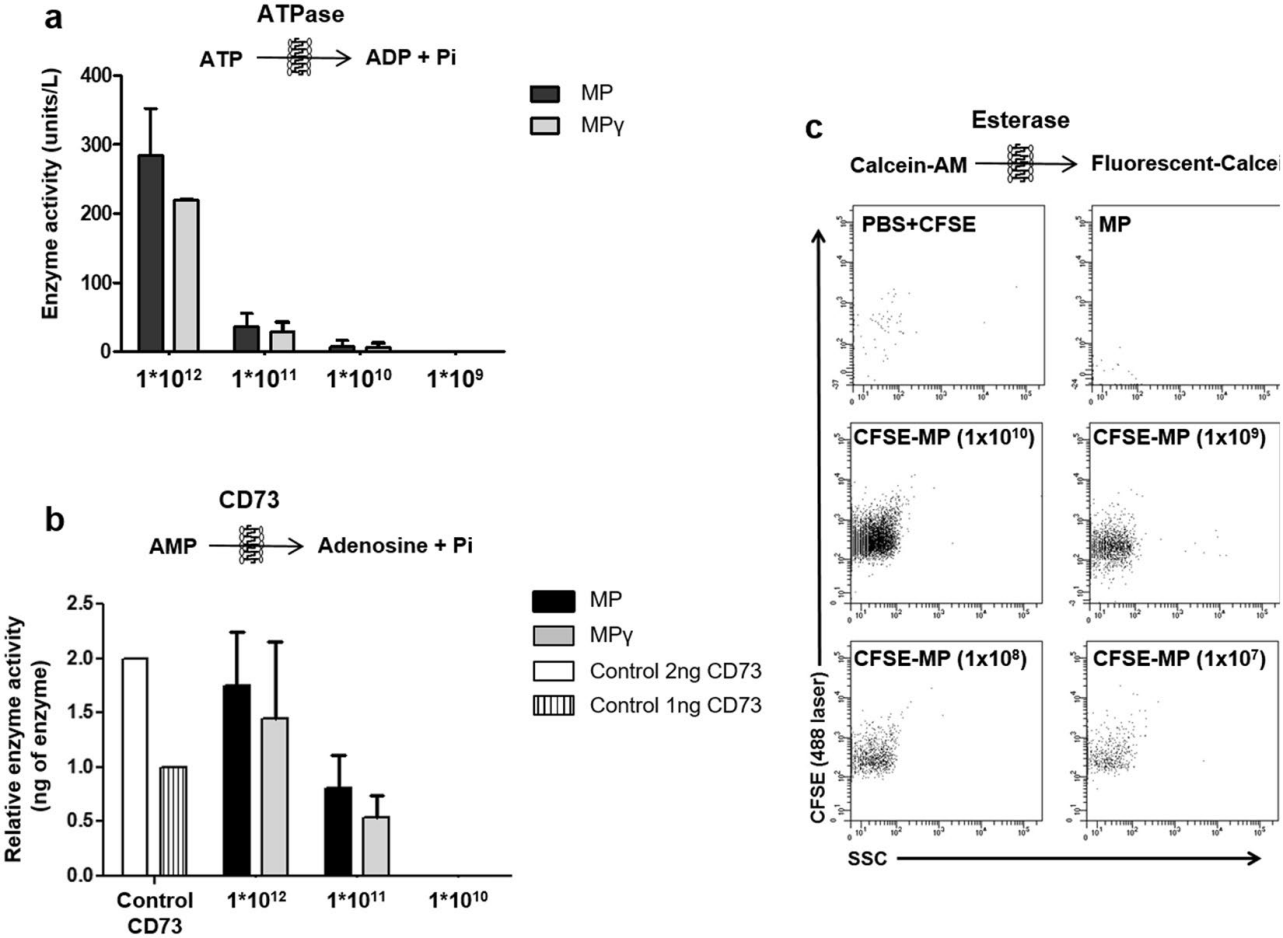
Enzymatic activity of Membrane Particles. (**a**) ATPase activity was measured at four different concentrations of MP (1 × 10^12^, 1 × 10^11^, 1 × 10^10^ and 1 × 10^9^ particles/ml). MP and MPγ were able to catalyze the breakdown of ATP and the detection of free phosphate was dependent on the concentration of MP. (**b**) The nucleotidase activity of the MSC marker CD73 was measured for three concentrations of MP (1 × 10^12^, 1 × 10^11^ and 1 × 10^10^ particles/ml). MP and MPγ were able to produce free phosphates after adding AMP substrate in a dose-dependent fashion. CD73 enzyme (2 and 1 ng) was used to calculate the concentration of CD73 in the MP. There was no statistical difference in enzyme activity between MP and MPγ. (**c**) Esterase activity of three concentrations of MP (1 × 10^9^, 1 × 10^8^ and 1 × 10^7^ particles/ml) was measured by the conversion of CFDA-SE to CFSE by flow cytometry. Fluorescent events were observed in MP labeled with CFSE (CFSE-MP), and the number of CFSE-MP detected was dependent on the concentration of MP. There was no statistical difference between MP and MPγ in esterase activity. Controls (PBS + CFSE and non-labeled MP) were negative. Data are presented as mean ± SD. Enzyme activities were detected in MP generated from 5 different MSC donors.

Figure 3a shows the ATPase activity (units/l) calculated from the standard curve generated with known free phosphate concentrations. MP and MPγ were able to convert ATP to free phosphate and the level of free phosphate was dependent on the concentration of MP. There was no statistical difference between MP and MPγ.

To examine whether MP and MPγ possess CD73 activity, the production of free phosphates by 2, and 1 ng of purified CD73 was compared with different concentrations of MP, and MPγ. Both types of MP were able to produce free phosphates after adding the substrate (AMP). The detection of free phosphate was dependent on concentration of MP and the amount of CD73 present in MP was calculated through the CD73 controls (Fig. 3b).

Esterase activity was measured by the conversion of non-fluorescent CFDA-SE to fluorescent CFSE by MP using flow cytometry based on a FITC fluorescence triggering strategy (Fig. 3c). This fluorescent-based flow cytometry protocol allows detection of particles based on positive fluorescence signals, not on size, as the average MP size of 120 nm is too small to be detected by most flow cytometers. Controls used for this flow cytometry protocol were PBS + CFDA-SE, and non-labeled MP (top 2 graphs). As expected, these controls were negative as no CFSE fluorescence can be expected. When MP were incubated with CFDA-SE, they converted CFDA to fluorescent CFSE, as demonstrated by the detection of fluorescent events (lower 4 graphs) showing that MP have esterase activity. As an additional control, MP were diluted before CFSE staining. The results shown indicate the recording of all samples during 1 min. The number of detected particles decreased for more diluted samples, but the MFI of the CFSE staining of the particles remained the same. This means that single MP can be detected with the used flow cytometry strategy. Fluorescent-based flow cytometry protocols were recently described in literature^29,30^.

### Effects of Membrane Particles on T cell proliferation

**Figure 4:**
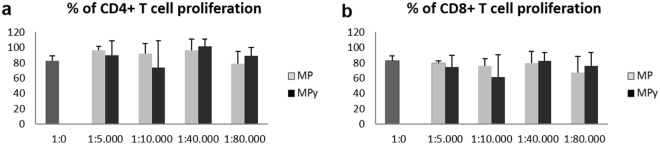
Effect of Membrane Particles on lymphocyte proliferation. CFSE loaded PBMC stimulated with anti-CD3/antiCD28 antibody were cultured with different ratios of MP for 4 days (1:5,000, 1:10,000, 1:40,000 and 1:80,000). CFSE dilution in CD4^+^ and CD8^+^ T cells was measured. (**a** and **b**) Addition of MP or MPγ did not affect the proliferation of CD4^+^ and CD8^+^ T cells. (n = 8; mean ± SD). Two-way ANOVA was used for statistical analysis.

CFSE loaded human peripheral blood mononuclear cells (PBMC) stimulated with anti-CD3/antiCD28 antibodies were cultured with different ratios of MP for 4 days (1:5,000, 1:10,000, 1:40,000, 1:80,000). To analyze lymphocyte proliferation, CFSE dilution was measured in CD4^+^ and CD8^+^ T cells. Addition of increasing concentrations of MP or MPγ did not affect the proliferation of CD4^+^ and CD8^+^ T cells (Fig. 4a and b).

### Membrane Particles decrease the proportion of CD16^+^ monocytes and increase CD90+ and PD-L1+ monocyte subsets

**Figure 5:**
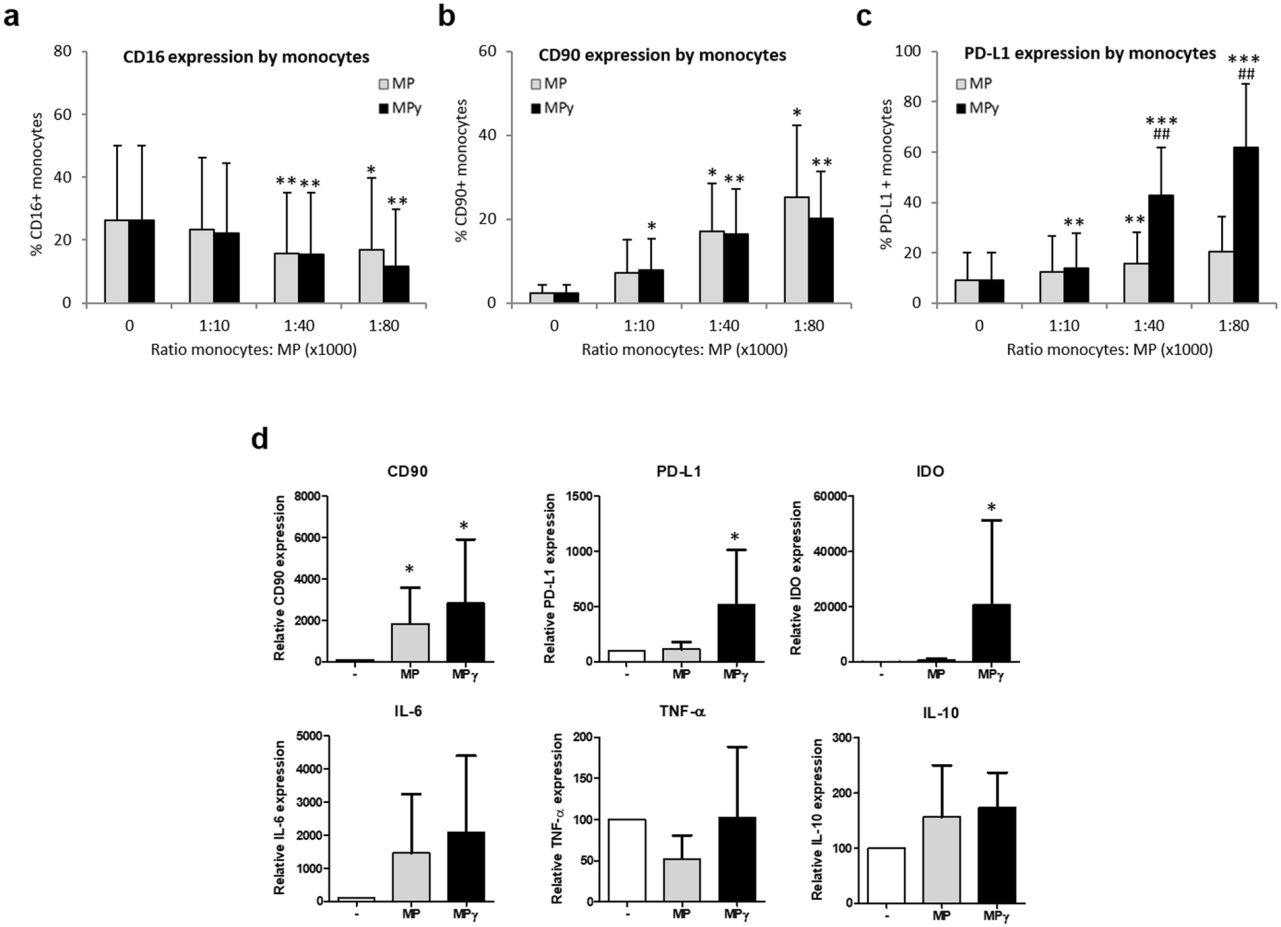
Effect of MP on CD14^+^ cells. Monocytes were cultured with different ratios of MP for 24 h (1:10,000, 1:40,000 and 1:80,000) to determine the effect of MP on monocyte immunophenotype. (**a**) Expression of CD16 on monocytes cultured in the presence of MP or MPγ (n = 6; mean ± SD). (**b** and **c**) Monocyte cell surface levels of CD90 and PD-L1 in the presence of MP or MPγ (n = 7; mean ± SD). (**d**) mRNA expression of monocytes after culture with MP. After 24 h of culture with MP or MPγ, monocytes were separated from MP and assessed by real-time RT-PCR for CD90, IDO, PD-L1, IL-6, TNF-α and IL-10 expression (n = 6; mean ± SD). Multiple comparison test (two-way ANOVA) was used for statistical analysis, *p < 0.05, **p < 0.01 and ***p < 0.001 vs control; ^#^ *p* < 0.05 and ^##^ *p* < 0.01 vs MP group.

Monocytes were cultured with different ratios of MP for 24 h (1:10,000, 1:40,000, 1:80,000) to determine whether MP could affect monocyte cell surface markers expression and immune function. Monocytes were cultured in polypropylene tubes to avoid the adherence of the cells and differentiation into macrophages. Culture of monocytes in the presence of MP or MPγ treatment decreased the frequency of pro-inflammatory CD14^+^CD16^+^ cells at ratios of 1:40,000 (by 45% and 49%, respectively) and 1:80,000 (by 48% and 35%, respectively) (Fig. 5a).

Monocytes treated with MP at ratios of 1:40,000 and 1:80,000 furthermore increased the expression of CD90 by 17% and 25%, respectively. Meanwhile, the MPγ group showed an increase in CD90 expression at ratios of 1:10,000 by 8%, 1:40,000 by 16% and 1:80,000 by 20% (Fig. 5b). Moreover, MPγ treatment induced anti-inflammatory PD-L1 expression in monocytic cells by 16% at a 1:10,000 ratio, 43% at a 1:40,000 ratio and 62% at a 1:80,000 ratio. MP had a smaller effect on PD-L1 expression with a 15% increase at a ratio of 1:40,000 (Fig. 5c).

### Membrane Particles affect the expression of pro- and anti-inflammatory genes in monocytes

In order to examine the effect of MP on monocyte immune function, and to examine whether the immunophenotypic changes observed were a result of protein transfer or of gene expression regulation, mRNA expression of a number of genes with pro- and anti-inflammatory function was analyzed in monocytes by qPCR after 24 h of stimulation with MP. Upregulation of CD90 gene expression as a result of particles stimulation was observed in MP and MPγ treated monocytes (*p* < 0.05) (Fig. 5d). Moreover, expression of the anti-inflammatory factors IDO and PD-L1 was increased in monocytes treated with MPγ, but not MP (*p* < 0.05) (Fig. 5d). There was a trend for increased expression of IL-6 after MP and MPγ treatment, but this was not significant. Significant changes in gene expression were also not observed for the pro-inflammatory cytokines TNF-α and anti-inflammatory cytokine IL-10.

### Membrane particles induce selective apoptosis of pro-inflammatory CD14^+^CD16^+^ monocytes

**Figure 6:**
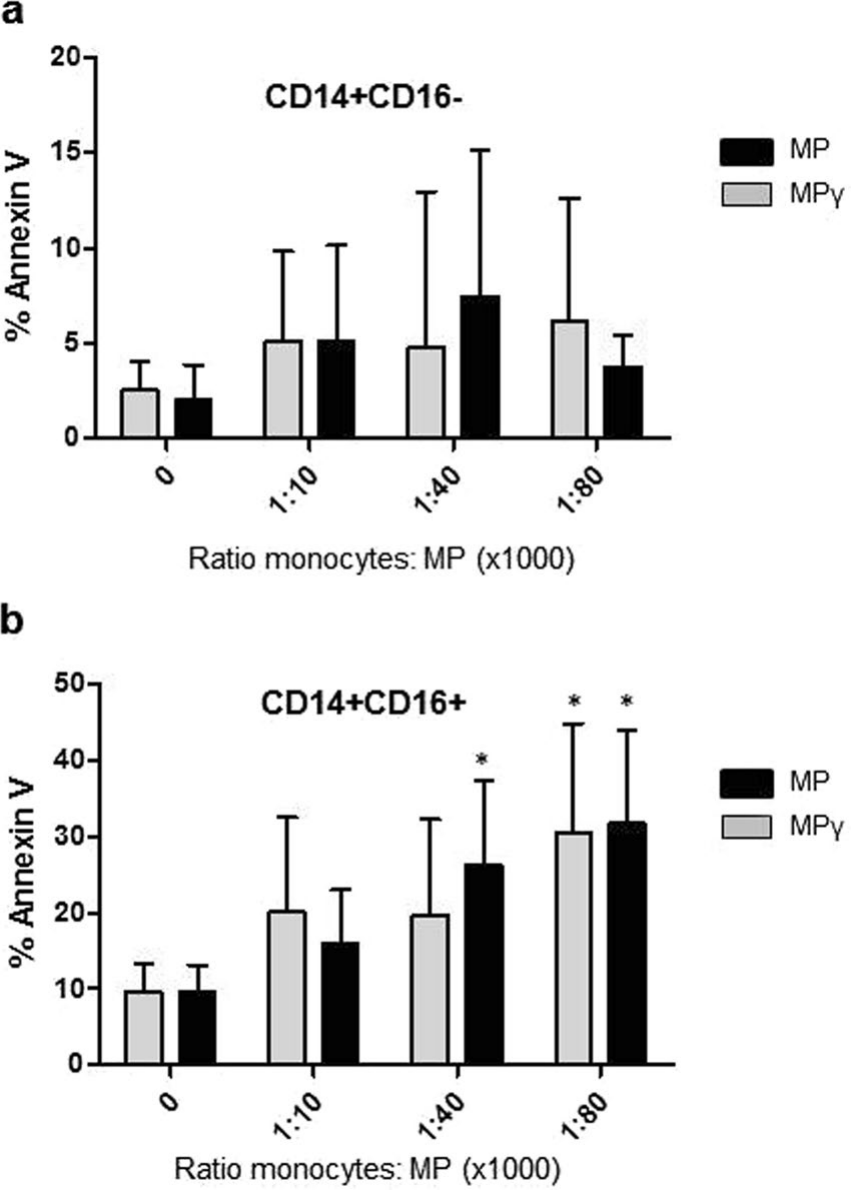
Effect of MP on apoptosis of monocyte subsets measured by Annexin V staining. Monocytes were cultured overnight with 3 ratios of MP or MPγ (1:10,000, 1:40,000 and 1:80,000). (**a**) Percentage of Annexin V positive CD14^+^CD16^−^ classical monocytes, and (**b**) percentage of Annexin V positive CD14^+^CD16^+^ pro-inflammatory monocytes. Data represent mean ± SD of 5 experiments using MP from 3 different donors. Two-way ANOVA was used for statistical analysis. P values (*p < 0.05) refer to the control without MP.

Monocyte incubated for 24 h with MP and MPγ (1:10,000, 1:40,000, and 1:80,000 ratios) were analyzed by flow cytometry for apoptosis by Annexin V staining. MP and MPγ did not significantly induce apoptosis in classical monocytes (CD14^+^CD16^−^) (Fig. 6a). However, pro-inflammatory monocytes (CD14^+^CD16^+^) showed an increase (*p* < 0.05) in apoptosis after incubation with MPγ at a ratio of 1:40,000, and after incubation of MP and MPγ at ratios of 1:80,000 (Fig. 6b). This indicated that MP specifically induce apoptosis of pro-inflammatory monocytes.

### Monocytes but not lymphocytes are able to take up Membrane Particles

**Figure 7:**
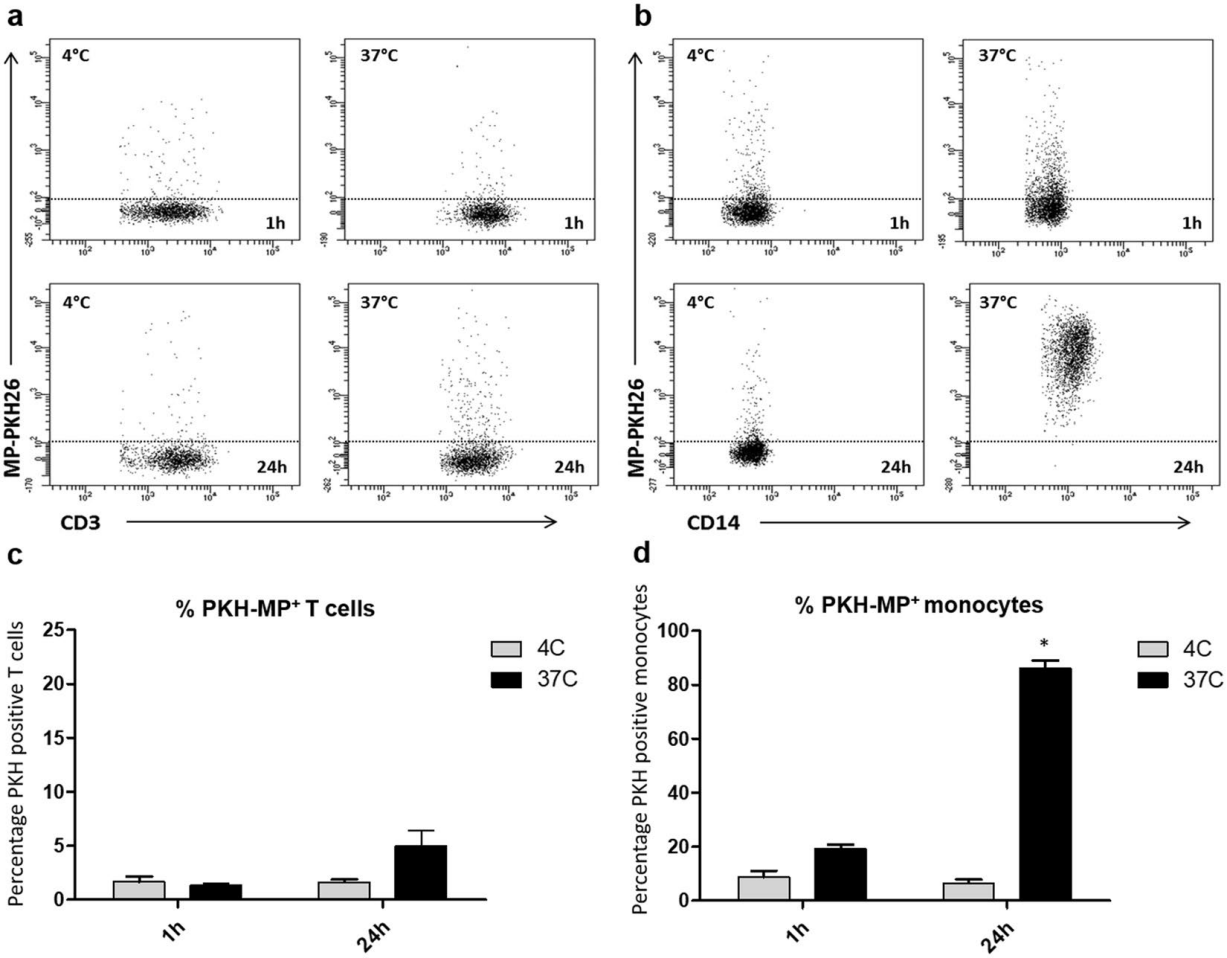
Uptake of MP by monocytes. MSC were labeled with PKH-26 before generation of MP (PKH-MP). PKH-MP were added to PBMC (ratio 1:40,000) and incubated for 1 h and 24 h at 37 °C. As a control the experiment was incubated at 4 °C. (**a** and **b**) Representative flow cytometry analysis of PKH-MP uptake by lymphocytes (CD3) and monocytes (CD14) at time points 1 h and 24 h at 4 °C and at 37 °C. (**c**) Percentage of CD3^+^ T cells positive for PKH-MP, and (**d**) Percentage of CD14^+^ monocytes positive for PKH-MP. Data are presented as mean ± SD from 6 experiments. Two-way ANOVA was used for statistical analysis. P values (*p < 0.05) refer to the 4 °C control at the 1 h time point.

Since the previous results showed that MP had immunomodulatory properties on monocytes but not on lymphocytes, we analyzed the interaction of MP with both types of immune cells. For that purpose, MP labeled with PKH membrane dye were added to PBMC (ratio 1:40,000) and incubated during 1 h and 24 h at 37 °C. As a control the cells were incubated at 4 °C, at which temperature no active uptake of MP is expected. A representative flow cytometry analysis is showed in Fig. 7a and b.

1 h after the addition of MP, a small percentage of CD3-lymphocytes (1.3 ± 0.2%) were positive for PKH-MP (Fig. 7c) while 20 ± 5.3% of CD14-monocytes was able to uptake MP (*p* < 0.05) (Fig. 7d). The difference between the MP uptake by monocytes and lymphocytes was higher after 24 h (lymphocytes: 5.2 ± 1.4%, monocytes: 93 ± 4.3%; *p* < 0.05). The 4 °C control for uptake was always below 3% for monocytes and lymphocytes in all the time points. This result indicated that MP uptake was mediated in an energy-dependent process.

**Figure 8:**
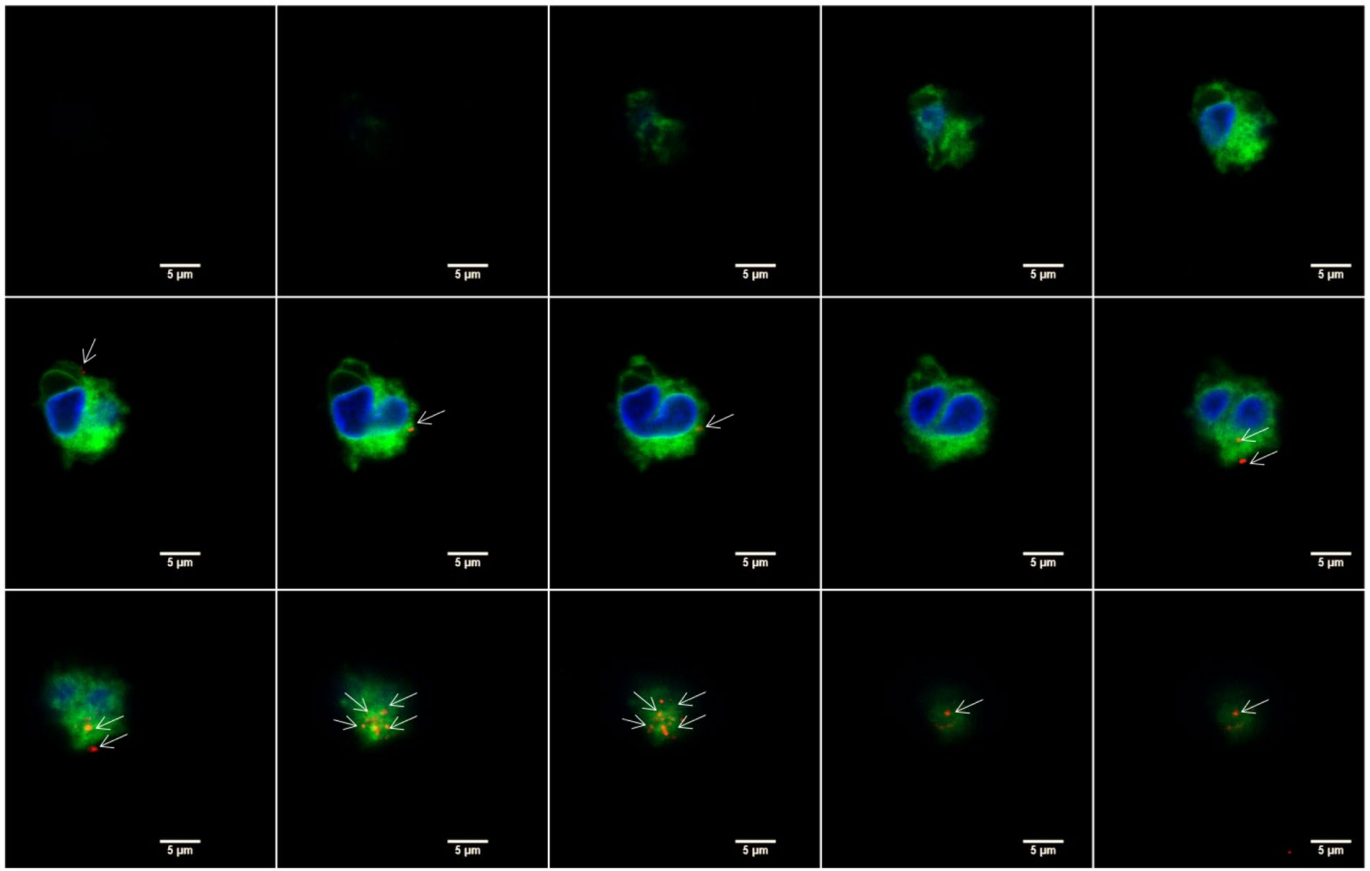
Confocal microscopy analysis of MP uptake by monocytes at 24 h. Z-stack images were collected at 1.2 μm intervals ranging from 0 to 17.6 μm. Staining for monocyte membrane (green), MP (red), and nucleus (blue) shows that MP are localized on the membrane of the monocytes (white arrows) and are not internalized. Scale bars: 5 μm.

To examine whether MP could be internalized by monocytes, confocal immunofluorescence microscopy was performed with isolated CD14^+^ cells from PBMC. The membrane of the monocytes was labeled with PKH-67 and cultured with PKH-MP (1:40,000). Time-lapse recordings showed that MP bound to the plasma membrane of the monocytes but they were not internalized. To look in detail at the localization of MP on the monocytes, z-stack images were analyzed by confocal microscopy (Fig. 8). These images confirmed that MP remained localized to the cell surface of the monocytes.

## Discussion

The immunomodulatory capacity of MSC is often attributed to the secretion of soluble factors^11^. We recently demonstrated that inactivated MSC without the capacity to secrete factors can modulate immune responses *in vitro* and *in vivo* ^9^. Inactivated MSC showed similar bio-distribution as living MSC as both are trapped in the lungs following intravenous administration. Here, we went one step further and generated nanoparticles from the membranes of adipose tissue MSC with diverse immunomodulatory properties by induction of regulatory proteins on the plasma membrane after treating the MSC with IFN-γ.

To generate MP, supernatants of MSC cultures were discarded and the cells were washed several times with PBS. Hereby the inclusion of soluble proteins in the MP preparations is avoided, which is a major challenge in the field of natural extracellular vesicles (EV) and causing misinterpretation of results^31^. The isolation methods for obtaining EV allow the co-precipitation of proteins, and RNA associated to lipoproteins secreted by the cells^32^. These contaminations mask the functional properties of EV and hamper their therapeutic application. With our novel protocol, we avoid the inclusion of artefacts from soluble molecules, and make MP a good alternative to EV.

Nanosight technology and electron microscopy were used for the characterization of MP. Most of the MP showed a size below 200 nm, and a round shape. Both characteristics make the MP an attractive therapeutic tool. Firstly, because their small size MP can easily maneuver through the capillary network of the lungs and reach sites of action beyond the lungs. Secondly, their morphology (closed circular structures) would allow loading of MP with compounds of interest and use MP as a delivery vehicle for future applications. Sun *et al*. provided evidence that anti-inflammatory drugs can be loaded into EV from myeloid cells and thereby enhance the delivery of the drug to activated monocytes in a LPS-induced septic shock model^33^. The use of MP from MSC as a natural delivery vehicle would have the advantage that the vehicle *per se* show immunomodulatory properties, which gives the carrier additional value. It is also important to consider that the production and manipulation of MP is easier and cheaper than the methodology used for the collection of EV, as it is possible to generate about 1.5 × 10^5^ particles/cell.

In addition to their morphological characteristics, MP were shown to possess enzyme activity. It has been reported that extracellular vesicles from MSC have a cargo rich in enzymatically active glycolytic enzymes, ATPases, and ATP-generating enzymes, such as adenylate kinase and nucleoside-diphosphate kinase^34^. Enzymatic activity has been demonstrated to be important for modulating the conditions in the vesicle nano-environment by consuming or generating metabolic energy. Katsuda *et al*. demonstrated the unique potential of extracellular vesicles from adipose tissue derived MSC for treatment of Alzheimer´s disease. These authors found that these extracellular vesicles carry Neprilysin, a metalloprotease, which ameliorates the disease’s symptoms^35^. We showed that MP possess nucleotidase and esterase activity, which are major enzymes regulating immunity and inflammation^21,22^.

Lymphocyte proliferation is the most commonly used assay to demonstrate the immunomodulatory capacity of MSC and it has been used as a standard assay to compare the immunosuppressive effect of MSC from different tissue sources. Comparative studies have sometimes however produced conflicting results. Puissant *et al*. have reported similar inhibition of T cell proliferation by bone marrow and adipose tissue MSC^36^, whereas Ribeiro *et al*. found that adipose tissue MSC to have stronger suppressive effects than bone marrow and umbilical cord MSC^8^. In pilot experiments, we generated MP from bone marrow derived MSC. These MP demonstrated similar properties as MP from adipose tissue MSC.

The mechanisms through which MSC suppress lymphocyte proliferation are largely dependent on soluble mediators. In our study, we found no effect of MP on lymphocyte proliferation. This can be explained by the fact that MP cannot secrete soluble factors, but also because lymphocytes were shown to be unable to bind or uptake MP. However, MP induced modulation of monocyte cell surface markers expression and changed their immune function. Furthermore, MP and MPγ induced the selective apoptosis of proinflammatory CD14^+^CD16^+^ monocytes.

CD16^+^ monocytes are major producers of inflammatory cytokines such as TNF-α and IL-12^37,38^, and high numbers of CD16^+^ monocytes are associated with acute and chronic inflammatory conditions^39^. Our results therefore suggest that MP act as immunomodulators that eliminate pro-inflammatory monocytes. Importantly, we also found that the immunomodulation induced by MP and MPγ is different. MPγ but not MP increased PD-L1 in the membrane of the monocytes and the mRNA expression of the anti-inflammatory factor IDO. Thus, the modification of the membrane protein composition of MSC by treatment of the cells with various stimuli provides us the opportunity to generate MP adapted for treatment of a specific immunological disorder. For example, MPγ with their enhanced capacity to induce PD-L1 and IDO by monocytes may be suitable for treatment of more severe immune responses involving inflammatory monocytes, while MP derived from MSC pre-treated with factors that induce proteins with regenerative function may be useful for inducing regenerative processes after resolving inflammation. As there is a lot of knowledge about modulation of MSC properties by cytokine treatment, there are tools in hand to control the make-up of MP. Thus, the potential therapeutic applications of MP are far reaching.

We demonstrated that the interaction of MP with monocytes is by binding and fusion with the plasma membrane of the monocytes. This is an active and specific mechanism for monocytes because at low temperatures MP were unable to fuse with the monocyte membranes. It is furthermore specific because MP do not bind to lymphocytes. The confocal microscopy images showed that there is no internalization of MP into monocytes, indicating phagocytosis plays no role in the uptake of MP. The mechanism of binding and fusion of MP with monocyte membranes supports the idea that MP can be a natural delivery vehicle for monocyte-targeting drugs.

**Figure 9:**
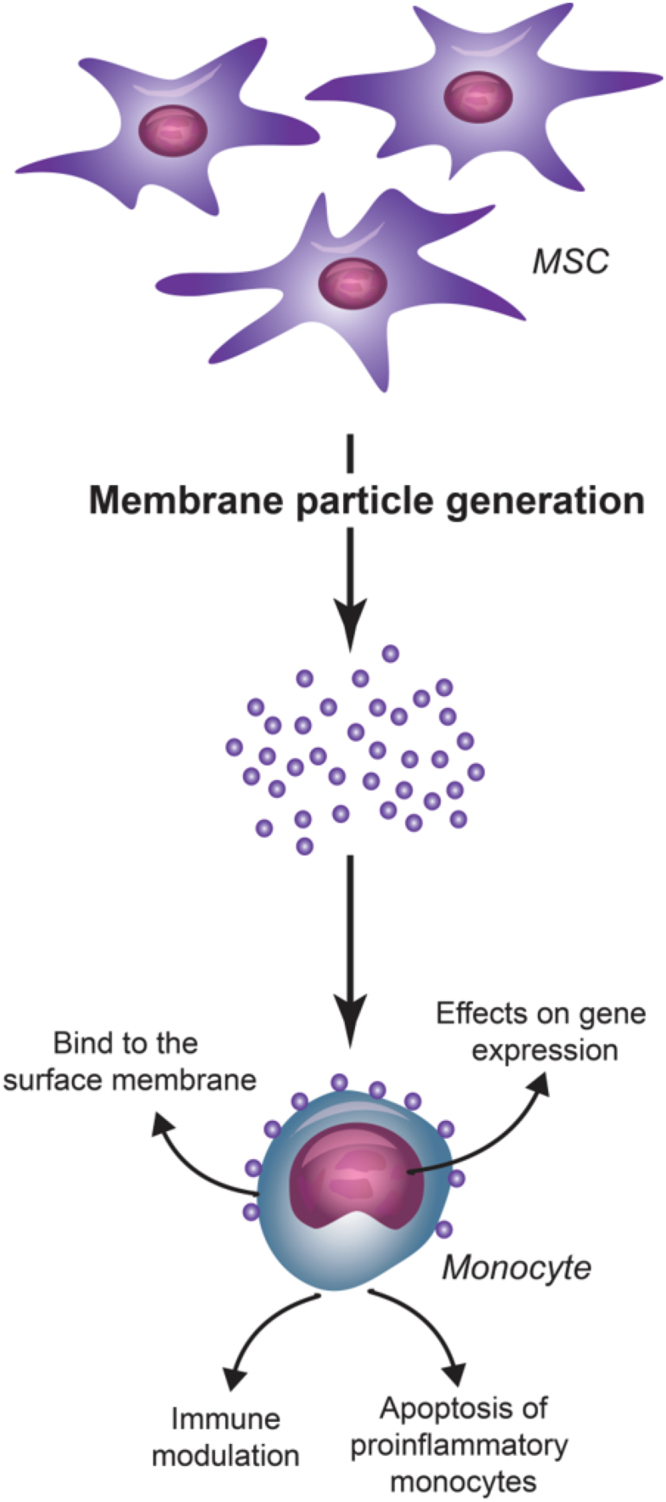
Schematic overview of the interaction of MP with monocytes. MP generated from MSC bind to monocyte plasma membranes. As an effect of the MP-monocyte interaction, MP modulate monocyte function by affecting gene expression and inducing apoptosis of pro-inflammatory monocytes.

In conclusion, MP represent a therapeutic strategy that combines the potential of MSC therapy with reduced risks associated with the use of living cells and improved ability to reach sites beyond the lungs. Our data demonstrates that MP target monocytes, via which they may have a broad immunomodulatory effect (Fig. 9). These data suggest that MP can serve as a novel cell-free therapeutic for treating immunological disorders. Additional studies, both *in vitro* and *in vivo*, are needed to improve our understanding the mechanisms of action of this potential immunosuppressive tool.

## Materials and Methods

### Ethics statement and human tissue samples

The MSC were provided by Internal Medicine Department, Transplantation laboratory of the Erasmus MC (The Netherlands). The cells were isolated from subcutaneous adipose tissue from healthy donors that became available during the kidney donation procedure. The tissues were not procured from prisoners, and were collected after obtaining written informed consent for all patients, as approved by the Medical Ethical Committee of the Erasmus University Medical Centre Rotterdam (protocol no. MEC-2006-190). All experiments were performed in accordance with the approved guidelines.

### Isolation and culture of MSC from adipose tissue

Subcutaneous adipose tissue from five healthy human kidney donors became available during the donation procedure. The adipose tissue was collected in minimum essential medium-α (MEM-α) (Sigma-Aldrich, St. Louis, MO) supplemented with 100 IU/ml penicillin, 100 mg/ml streptomycin (P/S) (Lonza, Verviers, Belgium), and 2 mM L-glutamine (Lonza).

The tissue was mechanically disrupted and enzymatically digested with 0.5 mg/ml collagenase type IV in RPMI for 30 min at 37 °C under continuous shaking. Thereafter, the cells were resuspended in MEM-*α* with 15% fetal bovine serum (FBS; Lonza), 2 mM L-glutamine and 1% P/S, filtered through a 100 µm cell strainer, and transferred to a 175 cm^2^ culture flasks (Greiner Bio-one, Essen, Germany).

Cultures were kept at 37 °C, 5% CO_2_, and 95% humidity, at 90% confluence; adherent cells were removed from culture flasks by incubation in 0.05% trypsin-EDTA (Life Technologies, Bleiswijk, The Netherlands) at 37 °C.

Two MSC culture conditions were used for the experiments: unstimulated MSC, and pretreated with IFN-γ (50 ng/ml, Sigma-Aldrich). After incubation for 3 days, MSC were collected to generate cell membrane particles. MSC were used for experiments between passages 2 and 6.

### Immunophenotypic characterization of AT-MSC

Unstimulated and IFNγ-stimulated AT-MSC were trypsinized and washed with FACS Flow (BD Biosciences, San Jose, CA). Cell suspensions were incubated with mouse-antihuman monoclonal antibodies against CD13-PE-Cy7; HLA-DR-PERCP; HLA-ABC-APC; CD31-FITC; CD73-PE; PD-L1-PE (all BD Biosciences); CD90-APC and CD105-FITC (R&D Systems, Abingdon, UK) at room temperature in the absence of light for 30 min. After two washes with FACS Flow, flow cytometric analysis was performed using FACSCANTO-II with FACSDIVA Software (BD Biosciences).

### Generation of cell Membrane Particles

Unstimulated and IFN-γ stimulated AT-MSC were trypsinized and washed twice with PBS. Then, the MSC were incubated in milliQ water at 4 °C until the cells exploded and liberated the nuclei (about 20 min). This step of the protocol was checked by microscopy. Then, the plasma membrane of cells was fractionated by passing them through a 29 G needle several times.

Cell extracts were cleared of unbroken cells and nuclei by centrifugation at 2,000 × *g* for 20 min. The obtained supernatant was transferred to an Amicon Ultra-15 100 kDa device and concentrated by centrifugation at 4,000 x *g* at 4 °C. The concentrated pellet consisted of crude plasma membrane and was diluted in 1 ml of 0.2 µm filtered PBS, cell culture medium or water. All procedures were performed on ice.

### Nanoparticle tracking analysis (NTA)

Analysis of absolute size distribution and concentration of MP was performed using NanoSight NS300 (NanoSight Ltd.). With NTA, particles are automatically tracked and sized based on Brownian motion and the diffusion coefficient. The analysis settings were optimized using as control filtered PBS and bovine serum albumin (BSA, Sigma-Aldrich) solution and kept constant between samples. The NTA measurement conditions were: detect threshold 3 (determined with the BSA solution), three measurements per sample (30 s/measurement), temperature 23.61 ± 0.8 °C; viscosity 0.92 ± 0.02 cP, frames per second 25. Each video was analyzed to give the mean, mode, median and estimated concentration for each particle size. The samples were diluted to obtain the right number of particles (1 × 10^8^ particles/ml) in accordance with the manufacturer’s recommendations.

### Transmission electron microscopy examination of MP

After fixation with paraformaldehyde (2%), all the samples were adsorbed for 20 min to glow-discharged carbon coated grids by floating the grids on 10 μL drops on parafilm. Grids with adhered MP were washed with water, stained with 2% uranyl acetate in water and examined in the electron microscope Tecnai T12 Spirit equipped with an Eagle CCD camera 4kx4k (FEI Company, Eindhoven, The Netherlands).

### ATPase assay

ATPase activity from MP and MPγ was measured using an ATPase assay kit according to the manufacturer’s instructions (Sigma-Aldrich). A phosphate standard was used for creating a standard curve. MP (1 × 10^12^, 1 × 10^11^, 1 × 10^10^ and 1 × 10^9^ particles/ml) were incubated with 4 mM ATP for 30 min at room temperature in assay buffer with malachite green reagent. The formation of the colorimetric product that formed in the presence of free phosphates was measured with a spectrophotometer at 620 nm.

As a control for possible phosphate contamination, the four MP concentrations were incubated in assay buffer without ATP. The signal from these samples was subtracted from the samples incubated with ATP.

### CD73 activity assay

A modified protocol of CD73 inhibitor screening assay kit (BPS Bioscience) was used to determine whether MP were able to degrade AMP into adenosine plus phosphate. MP and MPγ (1 × 10^12^, 1 × 10^11^ and 1 × 10^10^ particles/ml) were incubated with AMP (500 μM) during 25 min at 37 °C. Then, colorimetric detection reagent was added to measure the free phosphate from the CD73 reaction. Samples without AMP were measured as a control for free phosphate contamination. CD73 enzyme (2 and 1 ng) was used to calculate the concentration of CD73 in the MP, and MPγ.

### Esterase activity by CFSE

CFDA-SE, which is non-fluorescent, enters the cytoplasm of cells where intracellular esterases remove the acetate groups and convert the molecule to a fluorescent ester (CFSE). This conversion was used to detect whether MP have esterase activity. After MP generation, 1 × 10^10^, 1 × 10^9^, 1 × 10^8^ and 1 × 10^7^ particles/ml were labeled with 50 μM of CFDA-SE and incubated at 37 °C for 30 min. Dilution of the MP was performed to obtain a proper stoichiometry of the CFSE staining. PBS + CFDA-SE and non-stained MP were used as controls.

CFSE fluorescence was measured by flow cytometry (FACS Canto II, BD Biosciences). Due to the small size of the MP, reliable FSC and SSC measurements could not be obtained. Instead, MP were identified by setting a fluorescence threshold triggering on the CFSE fluorescence so that events above the threshold could be identified as CFSE-loaded MP.

### CD3/CD28 T cell proliferation assay

To evaluate the immunomodulatory capacity of MP, PBMC were labeled with 1 μM of CFSE and plated in round bottom 96-well culture plates at a density of 5 × 10^4^ cells/well. T cell proliferation was stimulated by adding human anti-CD3/anti-CD28 antibodies (1 µl/ml each) with a linker antibody Ig (2 µl/ml) (BD Biosciences). PBMC were incubated with different ratios of MP, or MPγ (1:5,000, 1:10,000, 1:40,000, 1:80,000) for 4 days. On the fourth day, non-adherent PBMC were removed from the plate, washed with FACS Flow and incubated with monoclonal antibodies against CD4-PerCP and CD8-PE-Cy7 (antibodies were purchased from BD Biosciences) at room temperature for 30 min. When a CFSE-labeled cell divides, its progeny are endowed with half the number of CFSE-tagged molecules and thus each cell division can be assessed by measuring the corresponding decrease in cell fluorescence by flow cytometry.

### Interaction of MP with monocytes

CD14^+^ cells were purified from PBMC using auto-MACS Pro by positive-selection. Monocyte purity was measured by flow cytometry after staining with mouse-antihuman monoclonal antibodies against CD14-PerCP (BD Biosciences) and CD3-PacBlue (BD Biosciences). Isolated CD14^+^ monocytes (2 × 10^5^ cells/200 µl) were cultured in RPMI 1640 medium (Life Technologies), supplemented with 10% FBS and 1% P/S. Monocytes were cultured with MP, or MPγ at different ratios (1:10,000, 1:40,000, 1:80,000) in polypropylene tubes. After 24 h of incubation, monocytes were collected for PCR analysis or flow cytometry after staining with CD14-PacBlue, CD3-PerCP, CD16-FITC, PD-L1-PE and CD90-APC (all BD Biosciences).

### Quantitative RT-PCR analysis

Monocytes were harvested, washed with PBS-diethylpyrocarbonate (DEPC; Sigma-Aldrich) and stored at −80 °C. Total RNA was isolated and 500 ng used for complementary DNA (cDNA) synthesis. Gene expression was determined by Quantitative Real-Time PCR (qPCR) using the TaqMan Universal PCR Master Mix (Life Technologies), and the assay-on-demand primer/probes for CD90 (Hs00264235_s1), PDL-1 (Hs00204257.m1), interleukin-6 (IL-6; Hs00174131.m1), IL-10 (Hs00174086.m1), tumor necrosis factor-α (TNF-α; Hs99999043.m1) (Thermo Fisher), and indoleamine 2,3-dioxygenase (IDO; Hs00158627.m1). Glyceraldehyde 3-phosphate dehydrogenase (GAPDH) mRNA served as housekeeping gene for normalization (Hs99999905.m1; Thermo Fisher).

### Apoptosis of monocyte subsets

Monocytes were cultured with MP, or MPγ at different ratios (1:10,000, 1:40,000 and 1:80,000) in polypropylene tubes overnight. Then, cells were incubated with monoclonal antibodies against CD14-Pacific Blue and CD16-FITC (antibodies were purchased from BD Biosciences) at room temperature for 30 min. After washing step, cells were stained with fluorochrome-conjugated Annexin-V for 15 min at RT to assess the apoptotic cells. All data were measured on a FACSCanto II flow cytometer (BD) and analyzed using FACSDiva software.

### MP uptake assays

To obtain fluorescent MP, MSC were labeled with the red fluorescent PKH-26 dye (PKH-MP), which intercalates into lipid bilayers, according to the manufacturer’s instructions (Sigma-Aldrich).

Human PBMC from healthy donors were isolated by density gradient centrifugation (Ficoll Isopaque, Sigma Aldrich) and cultured with PKH-MP (ratio 1:40,000). The incubation conditions were 37 °C, 5% CO_2_, and 95% humidity. As a control for the uptake process, PBMC were incubated with PKH-MP at 4 °C. PKH-MP uptake by lymphocytes and monocytes was analyzed by flow cytometry (FACS Canto II, Becton Dickinson) at 1 h, and 24 h.

Confocal microscopy analysis of PKH-MP uptake by monocytes was carried out by isolating CD14^+^ cells from PBMC using auto-MACS Pro by positive-selection (Miltenyi Biotec, Leiden, The Netherlands). Then, monocytes were labelled with PKH-67 (Life Technologies) and cultured with PKH-MP (ratio 1:4 × 10^4^) for 24 h. The nuclei of the monocytes were stained with DAPI. Images of monocytes were performed on a Leica TCS SP5 confocal microscope (Leica Microsystems B.V., Science Park Eindhoven, Netherlands) equipped with Leica Application Suite – Advanced Fluorescence (LAS AF) software, DPSS 561 nm lasers, using a 60 X (1.4 NA oil) objective. Images were processed using ImageJ 1.48 (National Institutes of Health, Washington, USA).

### Statistical Analysis

Data were analyzed for statistical significance either by Student’s t-test or one-way ANOVA analysis using GraphPad Prism 5 software. P < 0.05 was considered significant.

### Availability of data and materials

All data generated or analyzed during this study are included in this published article. The rest of datasets generated and/or analyzed during the current study are not publicly available due but are available from the corresponding author on reasonable request.

## References

[1] Bernardo, M. E. & Fibbe, W. E. Mesenchymal stromal cells: sensors and switchers of inflammation. *Cell Stem Cell* 13, 392–402, 10.1016/j.stem.2013.09.006 (2013). 10.1016/j.stem.2013.09.006 24094322

[2] Eggenhofer E, Luk F, Dahlke MH, Hoogduijn MJ (2014). The life and fate of mesenchymal stem cells. Front Immunol.

[3] Hao L (2012). Mesenchymal stromal cells for cell therapy: besides supporting hematopoiesis. Int J Hematol.

[4] Le Blanc K (2008). Mesenchymal stem cells for treatment of steroid-resistant, severe, acute graft-versus-host disease: a phase II study. Lancet.

[5] Reinders ME (2013). Autologous bone marrow-derived mesenchymal stromal cells for the treatment of allograft rejection after renal transplantation: results of a phase I study. Stem Cells Transl Med.

[6] Ciccocioppo R (2011). Autologous bone marrow-derived mesenchymal stromal cells in the treatment of fistulising Crohn’s disease. Gut.

[7] Luk F, de Witte SF, Bramer WM, Baan CC, Hoogduijn MJ (2015). Efficacy of immunotherapy with mesenchymal stem cells in man: a systematic review. Expert Rev Clin Immunol.

[8] Ribeiro A (2013). Mesenchymal stem cells from umbilical cord matrix, adipose tissue and bone marrow exhibit different capability to suppress peripheral blood B, natural killer and T cells. Stem Cell Res Ther.

[9] Melief SM, Zwaginga JJ, Fibbe WE, Roelofs H (2013). Adipose tissue-derived multipotent stromal cells have a higher immunomodulatory capacity than their bone marrow-derived counterparts. Stem Cells Transl Med.

[10] Lotfinegad P, Shamsasenjan K, Movassaghpour A, Majidi J, Baradaran B (2014). Immunomodulatory nature and site specific affinity of mesenchymal stem cells: a hope in cell therapy. Adv Pharm Bull.

[11] Hoogduijn MJ (2010). The immunomodulatory properties of mesenchymal stem cells and their use for immunotherapy. Int Immunopharmacol.

[12] Goncalves Fda C (2014). Intravenous vs intraperitoneal mesenchymal stem cells administration: what is the best route for treating experimental colitis?. World J Gastroenterol.

[13] Merino, A. *et al*. The Timing of Immunomodulation Induced by Mesenchymal Stromal Cells Determines the Outcome of the Graft in Experimental Renal Allotransplantation. *Cell Transplant*, doi:10.3727/096368917X695010 (2017). 10.3727/096368917X695010 PMC5657755 28160460

[14] Fischer UM (2009). Pulmonary passage is a major obstacle for intravenous stem cell delivery: the pulmonary first-pass effect. Stem Cells Dev.

[15] Schrepfer, S. *et al*. Stem cell transplantation: the lung barrier. *Transplant Proc* **39**, 573–576, 10.1016/j.transproceed.2006.12.019 (2007). 10.1016/j.transproceed.2006.12.019 17362785

[16] Eggenhofer E (2012). Mesenchymal stem cells are short-lived and do not migrate beyond the lungs after intravenous infusion. Front Immunol.

[17] Agrawal H (2014). Human adipose-derived stromal/stem cells demonstrate short-lived persistence after implantation in both an immunocompetent and an immunocompromised murine model. Stem cell research & therapy.

[18] Luk, F. *et al*. Inactivated Mesenchymal Stem Cells Maintain Immunomodulatory Capacity. *Stem Cells Dev*, doi:10.1089/scd.2016.0068 (2016). 10.1089/scd.2016.0068 27349989

[19] Vega-Letter AM (2016). Differential TLR activation of murine mesenchymal stem cells generates distinct immunomodulatory effects in EAE. Stem cell research & therapy.

[20] Sayed M (2014). Effects of Na/K-ATPase and its ligands on bone marrow stromal cell differentiation. Stem cell research.

[21] Chen X (2016). CD73 Pathway Contributes to the Immunosuppressive Ability of Mesenchymal Stem Cells in Intraocular Autoimmune Responses. Stem Cells Dev.

[22] Antonioli L, Pacher P, Vizi ES, Hasko G (2013). CD39 and CD73 in immunity and inflammation. Trends Mol Med.

[23] Moraes DA (2016). A reduction in CD90 (THY-1) expression results in increased differentiation of mesenchymal stromal cells. Stem cell research & therapy.

[24] Krampera M (2006). Role for interferon-gamma in the immunomodulatory activity of human bone marrow mesenchymal stem cells. Stem Cells.

[25] Ryan, J. M., Barry, F., Murphy, J. M. & Mahon, B. P. Interferon-gamma does not break, but promotes the immunosuppressive capacity of adult human mesenchymal stem cells. *Clin Exp Immunol* **149**, 353–363, 10.1111/j.1365-2249.2007.03422.x (2007). 10.1111/j.1365-2249.2007.03422.x PMC1941956 17521318

[26] English, K. *et al*. Cell contact, prostaglandin E(2) and transforming growth factor beta 1 play non-redundant roles in human mesenchymal stem cell induction of CD4 + CD25(High) forkhead box P3+ regulatory T cells. *Clin Exp Immunol* **156**, 149–160, 10.1111/j.1365-2249.2009.03874.x (2009). 10.1111/j.1365-2249.2009.03874.x PMC2673753 19210524

[27] Pourgholaminejad A, Aghdami N, Baharvand H, Moazzeni SM (2016). The effect of pro-inflammatory cytokines on immunophenotype, differentiation capacity and immunomodulatory functions of human mesenchymal stem cells. Cytokine.

[28] Trounson A, McDonald C (2015). Stem Cell Therapies in Clinical Trials: Progress and Challenges. Cell Stem Cell.

[29] Vendula Pospichalova *et al*. Simplified protocol for flow cytometry analysis of fluorescently labeled exosomes and microvesicles using dedicated flow cytometer. *J Extracell Vesicles* **4**, doi:10.3402/jev.v4.255302015 (2015). 10.3402/jev.v4.25530 PMC4382613 25833224

[30] van der Vlist EJ (2012). Nolte-‘t Hoen EN, Stoorvogel W, Arkesteijn GJ, Wauben MH. Fluorescent labeling of nano-sized vesicles released by cells and subsequent quantitative and qualitative analysis by high-resolution flow cytometry. Nat Protoc.

[31] Franquesa M (2014). Update on controls for isolation and quantification methodology of extracellular vesicles derived from adipose tissue mesenchymal stem cells. Front Immunol.

[32] Yuana, Y., Levels, J., Grootemaat, A., Sturk, A. & Nieuwland, R. Co-isolation of extracellular vesicles and high-density lipoproteins using density gradient ultracentrifugation. *Journal of extracellular vesicles* **3**, doi:10.3402/jev.v3.23262 (2014). 10.3402/jev.v3.23262 PMC4090368 25018865

[33] Sun D (2010). A novel nanoparticle drug delivery system: the anti-inflammatory activity of curcumin is enhanced when encapsulated in exosomes. Mol Ther.

[34] Clayton A, Al-Taei S, Webber J, Mason MD, Tabi Z (2011). Cancer exosomes express CD39 and CD73, which suppress T cells through adenosine production. J Immunol.

[35] Katsuda T (2013). Human adipose tissue-derived mesenchymal stem cells secrete functional neprilysin-bound exosomes. Sci Rep.

[36] Puissant B (2005). Immunomodulatory effect of human adipose tissue-derived adult stem cells: comparison with bone marrow mesenchymal stem cells. Br J Haematol.

[37] Merino A (2011). Senescent CD14 + CD16+ monocytes exhibit proinflammatory and proatherosclerotic activity. J Immunol.

[38] Zawada AM (2011). SuperSAGE evidence for CD14++CD16+ monocytes as a third monocyte subset. Blood.

[39] Merino A (2010). Effect of different dialysis modalities on microinflammatory status and endothelial damage. Clin J Am Soc Nephrol.

